# MicroRNAs miR-16 and miR-519 control meningioma cell proliferation *via* overlapping transcriptomic programs shared with the RNA-binding protein HuR

**DOI:** 10.3389/fonc.2023.1158773

**Published:** 2023-08-02

**Authors:** Sébastien Hergalant, Jean-Matthieu Casse, Abderrahim Oussalah, Rémi Houlgatte, Déborah Helle, Fabien Rech, Laurent Vallar, Jean-Louis Guéant, Jean-Michel Vignaud, Shyue-Fang Battaglia-Hsu, Guillaume Gauchotte

**Affiliations:** ^1^ INSERM, U1256, NGERE – Nutrition, Genetics, and Environmental Risk Exposure, Faculty of Medicine of Nancy, University of Lorraine, Vandoeuvre-lès-Nancy, France; ^2^ Department of Molecular Medicine and Personalized Therapeutics, University Hospital of Nancy (CHRU), Vandoeuvre-lès-Nancy, France; ^3^ Department of Biochemistry, Molecular Biology, Nutrition, and Metabolism, University Hospital of Nancy (CHRU), Vandoeuvre-lès-Nancy, France; ^4^ Department of Neurosurgery, University Hospital of Nancy (CHRU), Nancy, France; ^5^ CNRS, UMR7039, CRAN - Centre de Recherche en Automatique de Nancy, Université de Lorraine, Vandoeuvre-lès-Nancy, France; ^6^ Genomics and Proteomics, Department of Oncology, Luxembourg Institute of Health, Luxembourg, Luxembourg; ^7^ Department of Biopathology Institut De Cancérologie de Lorraine (CHRU-ICL), University Hospital of Nancy (CHRU), Nancy, France; ^8^ Centre de Ressources Biologiques BB-0033-00035, University Hospital of Nancy (CHRU), Nancy, France

**Keywords:** miR-16, miR-519, microRNA, HuR, proliferation, tumorigenesis, meningioma, transcriptomics

## Abstract

**Introduction:**

Meningiomas are the most common type of primary central nervous system tumors. In about 80% cases, these tumors are benign and grow very slowly, but the remainder 20% can unlock higher proliferation rates and become malignant. In this study we examined two miRs, miR-16 and miR-519, and evaluated their role in tumorigenesis and cell growth in human meningioma.

**Methods:**

A cohort of 60 intracranial grade 1 and grade 2 human meningioma plus 20 healthy meningeal tissues was used to quantify miR-16 and miR-519 expressions. Cell growth and dose-response assays were performed in two human meningioma cell lines, Ben-Men-1 (benign) and IOMM-Lee (aggressive). Transcriptomes of IOMM-lee cells were measured after both miR-mimics transfection, followed by integrative bioinformatics to expand on available data.

**Results:**

In tumoral tissues, we detected decreased levels of miR-16 and miR-519 when compared with arachnoid cells of healthy patients (miR-16: P=8.7e-04; miR-519: P=3.5e-07). When individually overexpressing these miRs in Ben-Men-1 and IOMM-Lee, we observed that each showed reduced growth (P<0.001). In IOMM-Lee cell transcriptomes, downregulated genes, among which ELAVL1/HuR (miR-16: P=6.1e-06; miR-519:P=9.38e-03), were linked to biological processes such as mitotic cell cycle regulation, pre-replicative complex, and brain development (FDR<1e-05). Additionally, we uncovered a specific transcriptomic signature of miR-16/miR-519-dysregulated genes which was highly enriched in HuR targets (>6-fold; 79.6% of target genes).

**Discussion:**

These results were confirmed on several public transcriptomic and microRNA datasets of human meningiomas, hinting that the putative tumor suppressor effect of these miRs is mediated, at least in part, via HuR direct or indirect inhibition.

## 1 Introduction

Meningiomas are the most common type of primary tumors of the central nervous system in adult. For brain meningioma alone, the annual incidence rate ranges from 1.3/100 000 to 7.8/100 000, a trend now under constant acceleration (1). WHO (World Health Organization) stratifies meningiomas into 3 grades of malignancy and 15 subtypes. These tumors originate from arachnoid cap cells forming one of the layers of the protective meninges, along with the dura and the pia mater, a membrane covering the brain and spinal cord. Regardless of grade, most patients undergo surgery if deemed adequate, but adjuvant therapy is not systematic because, to date, there is none validated for meningioma treatment (2). Plus, for grades 2 and 3, conformational radiotherapy is recommended after surgery (3). Thus, investigating the disease at a molecular level is an important issue as it may unlock new diagnostic and therapeutic options. However, it is only recently that meningioma genomic and epigenomic landscapes were described with enough accuracy to be helpful in precision medicine (2, 4, 5). By paving the way toward refined and clinically relevant classification systems (6–8), by fueling biomarker and drug target discoveries (9–12), these omic studies opened new areas of exploration to decipher the molecular characteristics of various meningioma subgroups (13–15).

MicroRNAs (miRs) are a class of 21–23 nucleotide-long non-coding RNA molecules involved in gene silencing and can modify gene expression at post-transcriptional level. They are of vital importance for the maintenance of balanced biological processes like cell proliferation and differentiation, metabolism, signaling, and death (16). Indeed, tissue-specific dysregulation of these miRs can trigger pathological consequences, and cancer. Interventions targeting abnormal miR expression account for effective treatment strategies for diverse diseases (17), offering alternatives with improved clinical outcomes (18). MiR-16, for example, constitutes a potentially useful biomarker for early detection in cancer diagnosis (19, 20) and an attractive therapeutic target (21, 22). Both miR-16 and miR-519 are dysregulated in several types of tumors (19, 20, 23–29), including glioma (30–32) and glioblastoma (33, 34). In human meningioma, however, neither the *in vivo* expression of these two miRs, nor their *in vitro* use as potential tumor suppressors have been evaluated, and to this day, few works have examined miR expression profiling in tumor tissue or serum of meningioma patients (35–40).

HuR (*ELAVL1*), a ubiquitously expressed RNA-binding protein involved in mRNA processing, stability, and transport, accounts for another promising drug target in anticancer treatment (41, 42). In meningioma, we previously described HuR overexpression as a marker of poor prognosis (43). Because miR-16 and miR-519 may negatively regulate HuR directly or indirectly (23, 25, 26, 44–46), we asked if restoration of these miRs in meningioma cells might reduce HuR and have anti-proliferative consequence. Therefore, the aims of this study were to determine whether miR-16 and miR-519 are differentially expressed in human meningioma relative to normal meningeal tissues, and to evaluate the effects of their overexpression on cell proliferation in human meningioma cell lines. Relative to healthy arachnoid tissues, we report miR-16 and miR-519 reduced levels in human meningiomas. Additionally, we explored the transcriptome-wide effects of miR-16 and miR-519 overexpression in high-grade meningioma IOMM-Lee cells and investigated the way these two miRs altered the expression of HuR and its target genes. Compared with our previous results on HuR transcriptomics and other human meningioma datasets of available miR profiling and transcriptome studies, these findings suggest that the putative tumor suppressor effect of miR-16 and miR-519 is mediated, at least in part, *via* HuR.

## 2 Materials and methods

### 2.1 Population and clinicopathological data

Sixty consecutive cases of intracranial grade 1 and grade 2 meningioma tissues were retrospectively retrieved from the Department of Pathology of the University Hospital of Nancy (institutional review board DC2008-459), and reviewed to confirm their initial diagnosis and grading according to the 2016 WHO classification criteria (47). Twenty samples of normal meningeal tissue were studied, including 10 samples of arachnoid membrane collected during autopsies and 10 surgical samples of non-neoplastic dura mater. Both meningioma and control tissues were fixed in formalin for 24 h.

### 2.2 Quantification of the relative expression levels of miR-16 and miR-519

In all tissue samples, the relative expression levels of miR-16 and miR-519 were determined *via* quantitative reverse transcription-polymerase chain reaction (qRT-PCR). Paraffin-embedded tumors and normal tissues were dissected from tissue blocks. Total RNA extraction was performed using TRIzol (Invitrogen, Life Technologies, Carlsbad, CA, USA). TaqMan MicroRNA Assays (Applied Biosystems, Foster City, CA, USA) were used for the quantification of miR-16 (hsa-miR-16, miRBase ID hsa-miR-16-5p, Applied Biosystems) and miR-519a (hsa-miR-519a, miRBase ID hsa-miR-519a-3p, Applied Biosystems), as previously described (24) and normalized against MiR-191 (hsa-miR-191-5p, Applied Biosystems), the reference microRNA in all experiments.

### 2.3 Cell lines

We used two cell lines, i) a human malignant meningioma cell line, IOMM-Lee cells (intraosseous malignant meningioma; a generous gift from Dr Gillespie and Dr Jensen, University of Utah, USA) (48), and ii) a benign grade 1 meningioma cell line, Ben-Men-1 cells, which were immortalized by retroviral transduction with human telomerase reverse transcriptase (Leibniz-Institut DSMZ-Deutsche Sammlung von Mikroorganismen und Zellkulturen GmbH, Germany) (49). The cell lines were cultured in Dulbecco’s modified Eagle’s medium (DMEM, Life Technologies, Carlsbad, California, USA) supplemented with 10% fetal bovine serum, 100 U/ml penicillin, and 100 mg/ml streptomycin at 37°C in 5% CO2.

### 2.4 MiR mimics transfection, cell growth, dose-response assays, and Ki-67 labeling index

The overexpression of miR-16 and miR-519 was achieved by transfection of mirVana miR Mimic hsa-miR-16-5p (Ambion, Life Technologies) and mirVana miR Mimic hsa-miR-519a-3p (Ambion, Life Technologies), respectively. MirVana miR Mimic Negative Control (Ambion, Life Technologies) was used as a control and referred throughout the work as miR-mimic negative control. Cell transfections, using Lipofectamine RNAiMAX Transfection Reagent, were performed following the manufacturer’s instructions. Transfection efficacy was verified 48h later using qRT-PCR technique.

For performing anchorage-dependent cell growth assay, 25,000 cells were incubated per well in 24-well plates. Cells were transfected with 17 nM of miR mimics. The number of cells per microliter was counted at 2, 4, and 6 days after transfection using LUNA Automated Cell Counter (Logos Biosystems, Annandale, USA). For performing dose-response assay, 10,000 cells were incubated per well in 48-well plates, 24 h before transfection. Cells were transfected with 0 nM, 0.17 nM, 1.7 nM, 3.4 nM, 17 nM and 170 nM of miRs. The number of cells per microliter was counted 96 h after transfection. Each measurement was performed three times after three independent transfections (n=9).

Additionally, cell proliferation was evaluated in IOMM-Lee cells based on the expression of Ki-67. The Ki-67 labeling index (LI) was evaluated 72 h after transfection, using anti-Ki-67 primary antibody (1/500; mouse monoclonal, MIB-1, Dako Cytomation), and fluorescent FITC anti-mouse Alexa Fluor (1/1000; Life Technologies) secondary antibody. A total of 500 cells in areas showing maximal nuclear intensity were used to compute the LI. Each measurement was performed three times after three independent transfections (n=9 in total).

### 2.5 Transcriptomics

Seventy-two hours after independent transfection with miR-16 (n=6, miR Mimic hsa-miR-16-5p), miR-519 (n=6, miR Mimic hsa-miR-519a-3p), and miR-mimic negative control (n=6, mirVana miR Mimic Negative Control), total RNA of the transfected IOMM-Lee cells was extracted using the TRIzol protocol (Invitrogen, Life Technologies, Carlsbad, CA, USA). Gene expression experiments were performed using the Affymetrix Human Gene v.2.0 ST Arrays according to GeneChip^®^ WT PLUS Reagent Kit, Manual Target Preparation for GeneChip^®^ Whole Transcript Expression Arrays P/N 703174 Rev.2 protocol; 100 ng of Total RNA were used as a starting amount for microarrays experiments; 3.5 µg of labeled DNA were injected into the Affymetrix cartridge. The arrays were hybridized with rotation at 60 rpm for 16 hours at 45°C. The arrays were washed and scanned according to the protocol GeneChip^®^ Expression Wash, Stain and Scan For Cartridge Arrays P/N 702731 Rev. 4.

Fluorescence values corresponding to raw expression data for each sample were extracted from each Affymetrix CEL files (one file per sample) using the R (v3.6) oligo package with the corresponding microarray platform definitions (pd.hugene.2.0.st). The extraction method included no normalization or background correction with the RMA algorithm. Positive and negative control probes were removed. The remaining 44.629 probes were annotated with up-to-date gene symbols using our local Ensembl database (version 83_38), allowing for accurate miR precursors and other ncRNA determination. Non-linear effects such as background or saturation were corrected by LOWESS normalization against a median profile of all samples (50). Data were then subjected to hierarchical clustering, which delineated clusters of co-expressed genes on one dimension and classified samples according to their expression profiles on another dimension. The method was applied on log2-transformed and gene-median-centered data, using uncentered Pearson’s correlation as similarity metric and average linkage to reconstruct the gene and sample dendrograms. Gene clusters were delimited by applying a distance threshold of 1/5 on the gene tree. Gene clusters separating control, miR-16 and miR-519 samples were then extracted, and a collective p-value (Student t-test) was computed between each group. For each sample, a mean expression value of all genes from the initial gene cluster was calculated and these values were compared between the selected groups. This strategy, based on strong correlation of gene expression, allowed us to avoid multi-testing as a means of p-value correction for the unsupervised analyses.

Differential gene expression analyses and statistics were achieved with moderated t-tests [linear modeling with empirical Bayes (51)] and corrected for the false discovery rate (FDR) with the Benjamini-Hochberg procedure. All clusterings were performed with Cluster 3.0 (52). For each identified gene list (gene cluster, gene signature, differential genes), functional annotations were performed using enrichR on multiple databases and gene sets (53). In-house enrichment analyses were conducted by calculating the ratio of frequencies Observed/Expected, where Observed was the frequency of the GO-Pathway-Disease term in the cluster or list, and Expected was the background frequency on the whole chip. Fisher’s exact tests were used to statistically validate the results.

MiR profiling and bulk-transcriptome public data were downloaded from the Gene Expression Omnibus (GEO, https://www.ncbi.nlm.nih.gov/geo/) database as raw gene datasets when possible, or processed datasets otherwise, and underwent the same quality control, preparation and annotation steps as described above.

### 2.6 Cell protein extraction and western blot

Seventy-two hours after IOMM-Lee cells transfection with miR-16, miR-519 and negative control miR mimics, total cellular proteins were extracted with RIPA buffer. Expression levels of HuR and GAPDH were then analyzed by Western blotting (n=9). The following primary antibodies were used: HuR (1/1000; rabbit polyclonal, Millipore), GAPDH (1/2000; chicken polyclonal, Millipore). Densitometry of all samples of Western blots were measured with Image J 1.42u (Wayne Rasband, National Institutes of Health, USA).

### 2.7 Statistical analyses

All quantitative variables are described as medians and percentiles [Interquartile range (IQR), 25–75th percentile]. All proportions are expressed as percentages with 95% confidence intervals (95% CI). Comparisons of miR-16 and miR-519 expression levels across the three tissue groups were performed using the Kruskall-Wallis test. Comparisons of miR-16 and miR-519 expression level of normal tissue and meningioma (both grade 1 and grade 2 subtypes) were carried out using the Mann-Whitney U test. When a statistically significant difference was found, the effect size estimated (r) for the difference between the two groups was calculated and interpreted according to Cohen’s method using z value. Cumulative probabilities of relapse-free survival were estimated by the Kaplan-Meier method. To evaluate the potential association between miR-16 and miR-519 and time to relapse, univariate analyses, using log-rank test were carried out on measures of miR-16, miR-519 and Ki-67 divided in medians. Log-rank tests were also performed using the online Cutoff Finder tool to screen for significant cutoff values (54). Cox proportional-hazard regression analysis was performed to identify independent variables predictive of relapse, using the following covariates: meningioma grade (1 or 2), miR-16, and miR-519 as continuous variables or as quartilized variables. Results were shown as hazard ratios (HRs) with 95% confidence intervals.

For serial measurements of cellular growth in the three experimental groups, we tested the change over time in cell viability and the difference between the three experimental groups over time using repeated measures analysis of variance (ANOVA) of log-transformed data. Two summary measures of interest were considered in serial analyses, namely: i) the area under curve considering the first value as the baseline value and ii) the percentage of the difference between the first and the last values. *Post-hoc* analysis for pairwise group comparisons was performed using the Student-Newman-Keuls test to avoid multiple testing issues. The measurement of progression of cell viability in the three experimental groups at successive times was carried out using the Friedman test for testing the difference between several related samples, as the same parameter was measured under different conditions in the same group. In the dose-effect study, three cell line groups were compared according to the type of miR transfected (miR-mimics for miR-16, miR-519, or negative control), and their concentration used which varied from 0 to 170 nM. The absolute number of viable cells at 96 h after the initiation of transfection was compared across the three groups by repeated measures ANOVA of log-transformed data. At each dose point, one-way Student t-tests were used to compare the number of viable cells between controls and miR-16 or miR-519.

## 3 Results

### 3.1 MiR-16 and miR-519 were underexpressed in human meningioma samples

**Figure 1:**
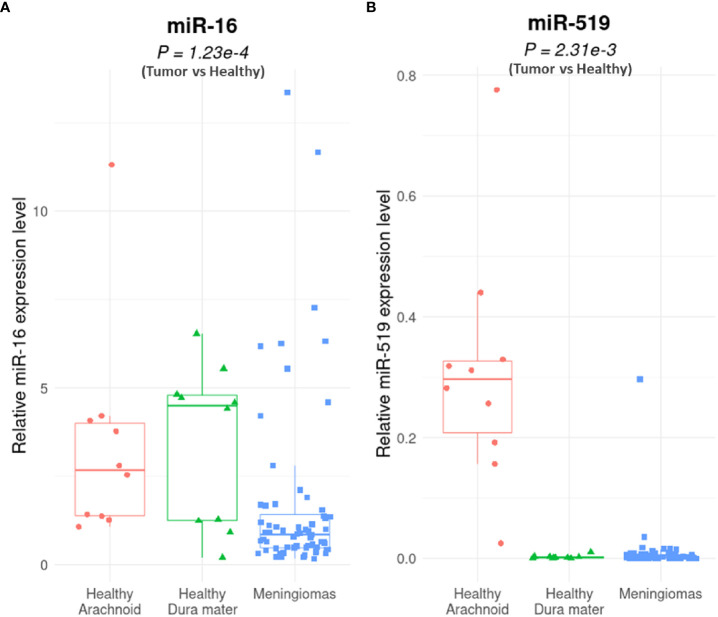
MiR-16 and miR-519 expression levels in human meningioma vs. healthy tissues. Quantitative reverse transcriptase polymerase chain reaction (qRT-PCR) analysis of miR-16 **(A)** and miR-519 **(B)** expression in human meningiomas (n = 64: 30 grade-1 and 34 grade-2) vs. non-tumoral tissue (n = 20: 10 arachnoid and 10 dura mater) (P = 1.23e-4 for miR-16 and P = 2.31e-3 for miR-519). Boxplot (minimum, first quartile, median, third quartile, and maximum) with in-dividual scatterplots.

**Figure 2:**
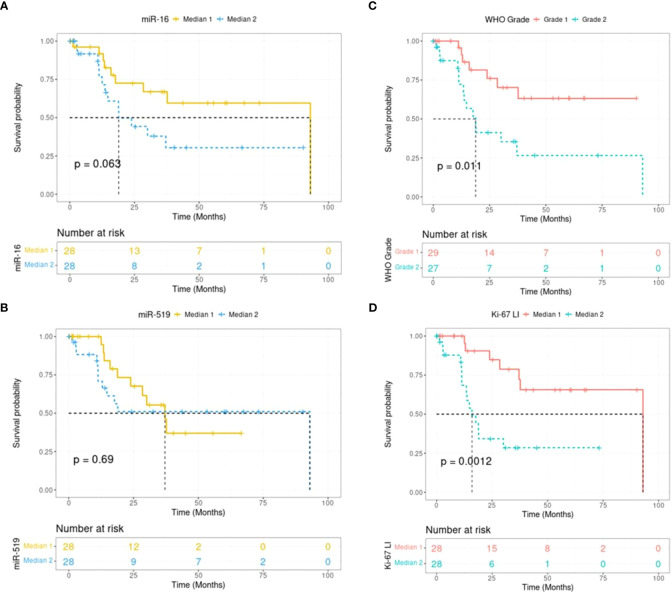
Kaplan-Meier plots showing recurrence-free survival in human meningiomas (n = 56). Survival curves are split in 2 groups for miR-16 **(A)**, miR-519 **(B)**, WHO grade **(C)** and Ki-67 **(D)**. Expression levels are grouped into medians, with Median 1 being the lower expression group. WHO, world health organization; LI, labeling index.

In patient meningioma samples (clinical data detailed in **Table 1**), lower levels of miR-16 were found in tumoral relative to control tissues (P = 1.23e-04; Mann-Whitney U-test), whether healthy arachnoid (P = 8.72e-04) or dura mater (P = 8.66e-03) (**Figure 1A**). MiR-519 expression was also lower in meningioma vs. control (P = 2.31e-03), with a clear differential against arachnoids (P = 3.52e-07) and no difference against dura mater (P = 0.76) (**Figure 1B**). In these samples, we found no significant difference in miR-16 and miR-519 levels between grade 1 and grade 2 tumors (**Figure S1**). Among this cohort, 22/56 (39%) patients were recurrence-free with the actuarial survival probabilities at their last known follow-up; neither the level of miR-16 nor that of miR-519 (expressed in medians) associated with post-surgical recurrence (**Figures 2A, B**). Positive associations were found between tumor grade, Ki-67 labeling index and higher risk of recurrence (P = 0.011 and P = 0.012, respectively) (**Figures 2C, D**), tumor grade and Ki-67 LI being highly correlated (rho = 0.74, P = 1.1e-10; Spearman’s test). Further analyses by the Cutoff Finder online tool (54) revealed that miR-16 and miR-519 showed no significant threshold (P = 0.07 and P = 0.20, respectively). Multivariate analysis using Cox proportional-hazards regression model consistently indicated that meningioma tumor grade was the only independent predictor of disease recurrence after adjusting for age, sex, miR-16, and miR-519 (P = 0.03).

**Table 1:** Summary of the demographic and clinical features of the patients with meningioma.

Features	WHO grade 1		WHO grade 2	
	(n = 32)		(n = 28)	
	%		%	
Gender				
Male	34%		61%	
Female	66%		39%	
Patient outcomes				
Death	0%		8%	
Recurrence	25%		60%	
Simpson’s grade				
Grade 1	44%		55%	
Grade 2	16%		17%	
Grade 3	40%		28%	
Treatment (excluding surgery)				
Radiation therapy	4%		44%	
Pre-operative embolization	8%		11%	
	**Median**	(IQR, 25^th^ – 75^th^)	**Median**	(IQR, 25^th^ – 75^th^)
Age, years (IQR, 25^th^ – 75^th^)	59	(54–70)	68	(57–72)
Follow-up duration, months	36	(12–55)	22	(11–46)

QR, interquartile range. Simpson’s grade (55): 1, macroscopically complete removal, including dura and bones; 2, macroscopically complete removal, dural coagulation; 3, complete removal, dura not coagulated. Grade: 2021 World Health Organization grading.

### 3.2 *In vitro* investigation of miR-16 and miR-519 in IOMM-Lee and Ben-Men-1 cells

#### 3.2.1 MiR-16 and miR-519 level in control and transfected cells

Given the results of decreased miR-16 and miR-519 in human meningioma against healthy tissues, we next examined the level of these miRs in two cultured meningioma cells lines, IOMM-Lee and Ben-Men-1 cells. We found that while miR-16 and miR-519 were nearly undetectable in IOMM-Lee cells, their levels were significantly higher in Ben-Men-1 cells (P = 2e-03 and P = 5e-03, respectively; **Figure S2**), with equivalent miR-16 and 5-times lower miR-519 levels in Ben-Men-1 as compared with healthy arachnoids. We proceeded to overexpress these miRs into both cell lines, noting that the transfection with miR-16 mimic did not significantly alter the expression of miR-519 (P = 1 in both IOMM-Lee and Ben-Men-1 cells), and vice versa (P = 0.13 in IOMM-Lee cells; P = 0.81 in Ben-Men-1 cells). The efficiency of transfection with miR-16 and miR-519 mimics was confirmed by qRT-PCR (**Figure S3**).

#### 3.2.2 Effect of miR-16 and miR-519 transfection on cell proliferation

**Figure 3:**
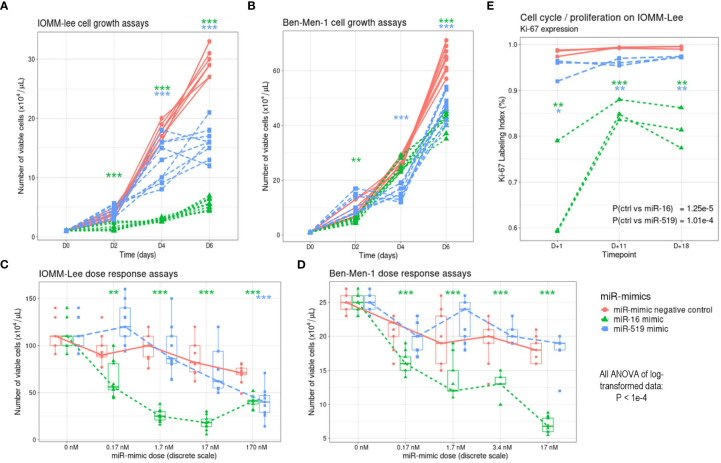
Effects of miR-16 and miR-519 overexpression in IOMM-Lee and Ben-Men-1 cells. **(A)** Meningioma cell viability measured in IOMM-Lee cells transfected with 17 nM of miR-16 and miR-519 mimics, and miR-mimic negative control (n = 9 in each group). **(B)** Similarly, meningioma cell growth measured in Ben-Men-1 cells. **(C)** Dose-response curve constructed by determining the number of viable IOMM-Lee cells 96 h after transfection with 0 nM, 0.17 nM, 1.7 nM, 17 nM and 170 nm of miR-16 and miR-519 (n = 9 in each group). **(D)** Similarly, dose-response curve obtained in Ben-Men-1 cells after transfection with 0 nM, 0.17 nM, 1.7 nM, 3.4 nM and 17 nm of miR-16 and miR-519. **(E)** Ki-67 expression at 3 different time points after transfection with 17 nM of miR-16, miR-519 and miR-mimic negative control (n = 3 in each group). *P < 0.05, **P < 0.01, ***P < 0.001 (one-way Student t test).

Cell growth of transfected IOMM-Lee cells was compared among three groups of cells, namely control (miR-mimic negative control), miR-16 and miR-519 mimics, on day 0, 2, 4, and 6 after transfection. Significant differences for both miRs were noted 2 to 4 days after miR transfection, with cells transfected with miR-16 showing the lowest growth (ANOVA of log-transformed data, P < 1e-04; pairwise comparisons, P < 0.05) (**Figure 3A**). Similar effects of the two miR-mimics were observed in Ben-Men-1 cells (both P < 1e-04) (**Figure 3B**). Ki-67 LI was lower in miR-16 (median, 81.4%; IQR, 72.9–85.2%) and in miR-519 (median, 96.4%; IQR, 95.8%–97.3%) miR-mimics transfected cells than in miR-mimics negative control (median, 99.2%; IQR, 98.8%–99.4%) transfected cells (P < 0.0001; *post-hoc*, P < 0.05 for all pairwise comparisons) (**Figure 3E**).

#### 3.2.3 Dose-response study of the effects of miR-16 and miR-519 on cell growth

In IOMM-Lee cell line, the number of viable cells was significantly lower in miR-16 transfected group than in both control and miR-519 transfected groups in all concentrations tested (0.17, 1.7, 17 and 170 nM) (all P = 1e-03) (**Figure 3C**), and the number of viable cells was significantly correlated with miR-16 transfection concentration (rho = -0.950; P < 1e-04). The number of viable cells did not differ between miR-519 and negative control-transfected groups, except at the highest dose (170 nM) where it was significantly reduced (P < 1e-04). Similar results were found in Ben-Men-1 cell line with the number of viable cells correlating significantly with the concentration of miR-16 (P < 1e-04) (**Figure 3D**). No significant inhibitory doses were found for miR-519 on this cell line.

### 3.3 MiR-16 and mir-519 transcriptomics

#### 3.3.1 Impact of the transfection with miR-16 and miR-519 mimics on the transcriptome of IOMM-Lee cells

**Figure 4:**
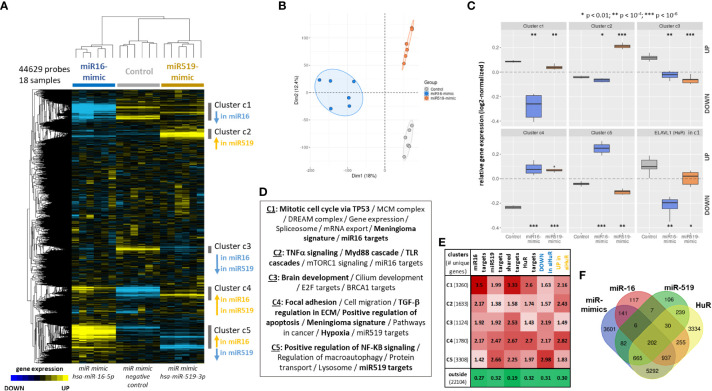
Effects of miR-16 and miR-519 transfection on the transcriptome of IOMM-Lee cells. A differential transcriptomic signature is extracted, dissected and compared for each miRNA (miR16: n = 6; miR-519: n = 6; negative controls: n = 6). **(A)** Hierarchical clustering heat map of the whole transcriptome delineating five significant clusters of co-expressed genes. Median-centered log2-transformed gene expression (arbitrary unit). Blue, yellow and black indicate downregulated, upregulated, and median genes, respectively. **(B)** Principal Component Analysis confirms the correlative structure within each group and the directional scattering between groups. **(C)** Boxplot comparing overall gene expression for the 5 significant identified gene clusters (c1 to c5) and for ELAVL1 (HuR). Student’s t-tests. **(D)** Top functional annotations associated with each gene clusters (with EnrichR, all q-values < 0.05). **(E)** Relative fold-enrichments computed for each five clusters (c1 to c5) and the rest of the microarray (outside) in target lists (from ENCORI https://starbase.sysu.edu.cn/ under high stringency): miR16 targets (1,695), miR519 targets (1,337), miR16 and miR519 overlapping targets (245), HuR targets (2,802; from Starbase v2), DOWN-regulated signature in siHuR transcriptome (4,725 genes), and UP-regulated signature in siHuR transcriptome (4,787 genes) (43). Fisher’s exact tests, all FDR < 0.01. **(F)** Venn diagram showing the overlaps between the miR-mimics signature comprising the five differential clusters and miR-16, miR519 and HuR targets (10,954; from ENCORI under high stringency). FDR: false discovery rate. * p < 0.01; ** p < 1e-04; *** p < 1e-06.

The transcriptome-wide effects of miR-16 and miR-519 transfection were analyzed in human anaplastic meningioma IOMM-Lee cell line (**Figure 4**). Hierarchical clustering was performed on three groups of samples: miR-16-mimics (n=6), miR-519-mimics (n=6) and miR-mimics negative controls (n=6). Each group was clearly separated from the other while retaining a high individual correlative structure (**Figure 4A**). These intergroup difference and intragroup cohesion were confirmed with principal component analysis, which illustrated the relative equidistance from controls and both miR-16 and miR-519 profiles, albeit on different axes of variances (**Figure 4B**). Shared features were also observed for the two miR-mimic groups. This segregation was driven by 5 clusters of strongly correlated genes (C1 to C5; **Figure 4A**), each of them differential vs. controls (**Figure 4C**), two of which displaying similar expression profiles for both miR-mimics (C3 and C4, down- and upregulated in both miR-16 and miR-519, respectively), the three others functioning in opposite directions (C1, downregulated in miR-16, C2, upregulated in miR-519, and C5, upregulated in miR-16 but downregulated in miR-519). These 5 clusters were functionally annotated (**Figure 4D**, **Tables S1–S5**) and associated with significant processes and pathways such as mitotic cell cycle *via* TP53, replication complexes (including MCMs) and gene expression *via* the DREAM complex (C1), TNFα signaling and immune response (C2), brain and cilium development (C3), regulation of apoptosis, hypoxia, cell migration, and the modulation of the extracellular matrix (C4), or NF-KB signaling and macroautophagy (C5) (all adjusted-P < 0.05). As expected, they were also highly enriched in miR-16 (mainly C1 with 34%) and miR-519 (mainly C5 with 26.5%) mRNA targets (**Figures 4D, E**), and in meningioma signature genes (C1 and C4). Multivariate statistics confirmed these results and yielded 510 differential genes between miR-16 and control transcriptomes, and 152 genes between miR-519 and controls (FDR < 0.05, differential > 2-fold-change; moderated t-tests on linear modeling with empirical Bayes; **Tables S6**, **S7**). Few of these top genes overlapped between miR-16 and miR-519 (35 unique genes up in both miR-mimics, 2 unique genes down in both miR-mimics, 2 unique genes down in miR-16 while up in miR-519).

#### 3.3.2 Transcriptome-wide effect of miR-mimics on HuR (*ELAVL1*)

Next, we asked if and how these five differential and functional clusters obtained with miR transcriptomics (C1 to C5) were enriched in HuR targets and in the HuR transcriptomic signature previously identified by our group in a similar setup on meningioma samples with HuR knockdown (GSE95212, 43). Compared to cells expressing miR-mimic negative control, both miR-16 and miR-519 transfected cells had significantly lowered level of *ELAVL1* (HuR) mRNA (FDR = 6.1e-06 and FDR = 9.38e-03, respectively; moderated t-tests; **Figure 4C**). Western blot showed that the expression level of HuR was significantly lower (2.2-fold decrease) following the transfection with miR-16, but not miR-519 mimics (P < 1e-04 and P = 0.8, respectively). Remarkably, *ELAVL1* was one of the downregulated genes of cluster C1 with functions in pre-replicative complex and cell cycle. Further gene enrichment analysis showed that 79.6% of known HuR targets [2802-gene list obtained from Starbase v2 https://starbase.sysu.edu.cn/starbase2/ for HuR transcriptomic compatibility (43)] were distributed in the five clusters, the most represented being C1 (26.1%; 2.6-fold enrichment), C4 (15.9%; 2.7-fold enrichment) and C5 (21.4%; 2-fold enrichment) (all P < 2.2e-16; Fisher’s exact test; **Figure 4E**). Furthermore, genes that were dysregulated in our HuR knockdown transcriptomic experiment were also found enriched in the present miR-mimics signature. Downregulated genes in HuR knockdown mainly distributed in clusters C3, C4 and C5 (5.5%, 8.7% and 22.1%; 2.2, 2.2 and 3-fold enrichment, respectively). Conversely, genes that were upregulated by HuR knockdown distributed in C1, C2 and C4 (15.8%, 8.9% and 11.2%; 2.2, 2.4 and 2.8-fold enrichment, respectively). Genes outside the miR-mimics signature were 3-times depleted in HuR knockdown genes (all P < 2.2e-16; **Figure 4E**).

#### 3.3.3 MiR-mimics signature of shared miR-16, miR-519 and HuR targets

Considering the high levels of enrichment of the five transcriptomic clusters in miR-16 or miR-519 target mRNAs (**Figure 4E**), we consolidated this large 5-cluster signature into a more informative feature containing only 208 shared targets of both miRs. Nearly all genes (202/208) were also HuR targets (**Figure 4F**). Given their differential expressions and their combined functions, the redistribution of these genes back into C1 to C5 can be considered highly representative of the larger signature (**Figure S4**, **Table S8**). Indeed, the 208-gene signature contained both transcriptional regulators involved in cell cycle (such as E2F genes) and protagonists of proliferation (*MKI67*, *CCND2*, *CDKN1A*). The signaling network reconstructed with these 208 genes linked them functionally around meningioma relevant hubs such as AKT3, CDKN1A, PAK2 and PRKAA1 and revealed functions and pathways including cell cycle progress, differentiation, DNA damage response, mRNA nucleus export, growth factors and fibrosis (all FDR < 0.05; **Figure S4**).

#### 3.3.4 The miR-mimics signature differentiates between subgroups of meningiomas with distinct proliferative features

Finally, we evaluated our findings on relevant public datasets of human meningiomas. On a miR profiling dataset [GSE126563 (40)] of primary (n=44) and secondary (n=15) tumors we found diminished miR-16 levels when compared to controls (n=5; P = 0.047 vs. all tumors, and P = 0.035 vs. primary meningiomas alone). On another dataset of various meningioma grades [GSE50641 (36)], we found lower miR-16 expression in grade 2 (n=11) as compared to grade 1 (n=33), which already displayed very low levels (P = 0.037). In either set, miR-519 was below detection and could not be tested.

**Figure 5:**
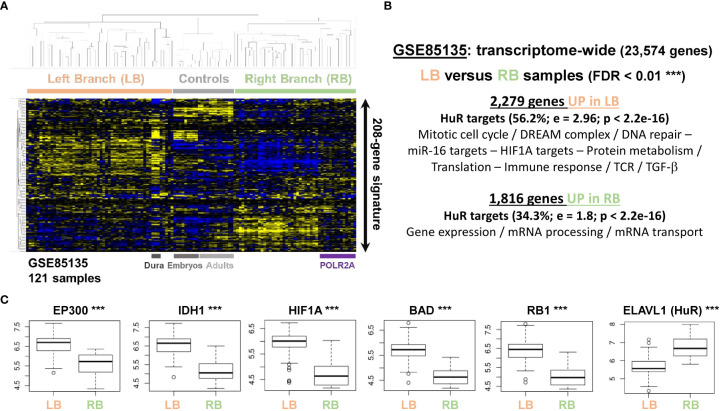
The miR transcriptomic signature distinguishes two subgroups of samples with different proliferative features in a landmark dataset of grade 1 meningiomas (GSE85135 (14)). **(A)** Unsupervised hierarchical clustering according to the reduced 208-gene miR-16/miR-519 signature. **(B)** Transcriptome-wide differential statistics between the 2 groups of samples identified with the reduced signature (LB, RB). Up- and downregulated gene lists were functionally annotated with EnrichR (adjusted p-values < 0.05). Control samples were not included in the statistical analyses. **(C)** Boxplots representing the differential expressions between LB and RB for a select list of genes reported to be consistent markers of meningioma recurrence and aggressiveness, and HuR. These genes do not belong to the 208-gene signature. Y-axis: normalized gene expression (log2). Enrichment in HuR targets: Fisher’s exact test; e, relative fold-enrichment. LB, left branch of the dendrogram; RB, right branch of the dendrogram. *** FDR < 0.01.

We also investigated a landmark transcriptome of 121 meningiomas [GSE85135 (14)], where the miR-mimics signature highlighted two dominant clusters with different expression profiles, named left and right branch (LB and RB, respectively) of the clustering tree (**Figure 5A**). According to this 208-gene hierarchical clustering, healthy controls expectedly displayed a correlated pattern for both embryologic and adult tissues, distinct from LB and RB profiles. Interestingly, the dura mater control samples clustered preferentially with tumoral samples on the LB, instead of regrouping with the rest of the controls. Investigating on these profiles from the whole transcriptome led to the same conclusion, with little to no overlap with the adult arachnoids. The overlap with tumoral and embryologic tissues was enriched in miR-519 targets (>1.8-fold; P < 2.2e-16), confirming our findings in human tissues (**Figure 1B**). After removing controls, transcriptome-wide differential statistics between LB and RB respectively yielded 2,279 and 1,816 up- and downregulated genes in LB (FDR < 0.01, **Figure 5B**), which amounted to 18% of the measured genes. Remarkably, these genes were extremely enriched in HuR targets, 56.2% of which were UP in LB (DOWN in RB) and 34.3% UP in RB (or DOWN in LB). Genes UP in LB revealed functions linked to mitotic cell cycle, DNA repair and immune response and were also enriched in miR-16 and HIF1A targets. Genes UP in RB were associated to mRNA processing and transport (all FDR < 0.05). Moreover, we checked *EP300* level, which was reported to be a solid marker of meningioma recurrence, independently of WHO grade (11). Consistent differences were observed between the two subgroups (P = 2.75e-10; **Figure 5C**) for this mRNA, along with other markers associated with aggressiveness/proliferation, also outside the 208-gene list (*HIF1A*, *RB1*, *BAD* or *IDH1*; all FDR < 1e-14). Furthermore, *ELAVL1* (HuR) showed reduced levels in the subgroup with diminished gene expression and mRNA transport processes and was overexpressed in the subgroup with increased cell cycle and proliferation features (FDR = 3.76e-04). On another transcriptomic dataset [GSE74385 (9)], the miR-mimics signature again classified samples in two groups. WHO grade 1 (13/16) and non-recurrent (16/20) meningioma were over-represented in the first, the second regrouping higher grades (29/37) and recurrent tumors (14/16), with decreased *EP300* (P = 1.13e-04) and increased *FOXM1* (P = 6.62e-07) levels, previously reported in recurrent meningiomas, as down- and upregulated, respectively (11).

Finally, to validate our main transcriptomic findings, we performed an integrative analysis of a multi-omic meningioma dataset with bulk transcriptome coupled with miR measurements for the same patients (GSE88721), including samples of heterogeneous histological origin and grades (1 and 2) (56). Focusing on miR-16 and miR-519 mature products, along with their premature forms, we associated the miR expression levels with that of hallmark target genes representing the whole correlated panorama (**Figure S5**). Expression of *ELAVL1* was found negatively correlated with miR-519 (r2 = -0.69; Pearson’s correlation) but not with its premature forms (r2 = 0.38), potentially indicating a preferential miR-519 inhibition of *ELAVL1* transcripts in meningiomas, rather than the inhibition of miR-519 by HuR and/or as a consequence of other transcriptomic changes. The hypoxia factor gene *HIF1A* correlated with both miR-16 and miR-519 levels (r2 < -0.4, respectively). G1/S Cyclin D3 (*CCND3*), from the pre-replicative complex and cell cycle progression marker, was found significant with both miRs, along with *RB1* disease progression marker (and directly inhibited by Cyclin D3 upon phosphorylation), strongly associated with miR-519a (r2 = -0.62). In line with our transcriptomic clusters, we also report *BCL2L1* (potent apoptosis regulator and caspase inhibitor) and *NFKB1* associations with miR-519, and *MTOR* and *TNF* associations with miR-16.

## 4 Discussion

MicroRNA expression is an open subject in human meningioma where limited number of studies have explored tissue and serum samples (2). For example, Zhi et al. found increased serum content of miR-106a-5p, miR-219-5p, miR-375 and miR-409-3p, and decreased content of miR-197 and miR-224 in meningioma patients (37). The putative functions of these miRs seem to depend on their relative expression level in tumor vs. normal tissue. For instance, the overexpression of miR-335 in meningioma samples was used to evidence its role as “oncomiR” (57), while the downregulations of miR-200a and miR-145 were used to support their tumor suppression function (58). Likewise, in a retrospective study, higher miR-190a expression level was reported to be an independent prognostic factor of meningioma recurrence rates and lower miR-29c-3p and miR-219-5p were found to be associated with advanced clinical stages of meningioma (35). Ludwig et al. identified several dysregulated miRs between different subtypes of benign meningiomas, and in anaplastic vs. benign tumors. They further marked a 4-miR signature, miR-222, miR-34a, miR-136, and miR-497, as differentiating WHO grade 2 from grade 1 meningiomas in a setup of 55 samples of various histological types (36). MiR-34a was next confirmed as differential between grade 2 and grade 1 meningiomas in a study led by another group (38). However, no other reported miR otherwise overlapped with the previous works, suggesting that larger cohorts of patients might be needed to overcome the heterogeneity of meningioma subtypes. More recently, miR-15a, miR-146a, and miR-331 were identified as good prognosticators of relapse (40), in a design of paired primary vs. recurrent tumors and a large validation cohort. However, they did not validate the previously reported miR-190a as differentially expressed. Conversely, Negroni et al. confirmed miR-497 as a circulating biomarker for high-grade meningiomas, with lower levels in serum exosome samples as compared with benign meningiomas (39). Here we found both miR-16 and miR-519 downregulated in benign and atypical meningioma vs. normal arachnoid tissues.

MiR-16 and miR-519 have previously been shown to be tumor suppressor miRs in several types of tumors, including laryngeal squamous cells, non-small cell lung carcinoma, breast carcinoma, nasopharyngeal carcinoma, prostate cancer, glioma and glioblastoma (23–34, 59), but were not investigated as such in meningioma. We found them both downregulated in human samples. This result was replicated for miR-16 in a miR dataset of healthy tissues and primary + secondary tumors (40), albeit only as a trend probably because of the limited number of controls samples (P < 0.05). On another meningioma dataset (36), we found diminished miR-16 levels in grade 2 versus grade 1 tumors. This trend (P < 0.05) should be validated in larger and more homogeneous cohorts given that benign tumors were subdivided into meningothelial, fibroblastic and transitional meningioma variants. In our data we did not find significant change of miR-16 levels between grades. Moreover, in all available data miR-16 expression was already very low in grade 1, making any comparison between grades hazardous. For miR-519, no exploitable data was available as expression reached background levels in every dataset. In-depth sequencing could solve these problems and thus appears to be a much-needed endeavor in meningioma miR profiling. Especially because very few miR datasets are publicly available and/or computationally exploitable.

In addition to their downregulation in human tissues, here we studied the consequences of miR-16 and miR-519 overexpression in benign as well as malignant cell lines. We found both miRs tumor-suppressive. In addition, we show that the repressive effect of miR-16 is dose-dependent, like what was reported by Reid et al. in malignant pleural mesothelioma cells (60). Interestingly, this cell growth inhibition was much less pronounced in the benign Ben-Men-1 cell line than in the anaplastic meningioma IOMM-Lee cell line, probably since IOMM-Lee cells have lower basal level of miR-16 and miR-519 than Ben-Men-1 cells, resulting in their higher sensitivity to the overexpression of these miRs. Confirming the growth inhibition on another cell line is therefore necessary before drawing conclusions. Nevertheless, our findings suggest that miR-16 and miR-519 mediate anti-tumorigenic processes *via* inhibition of cell proliferation. A fact corroborated by lower Ki-67 labeling index in IOMM-Lee cells overexpressing either miR-16 or miR-519.

The results of the transcriptomic study also suggest that the cell growth inhibition by either miR is mediated by downregulations of both the pre-replicative complex and cell cycle *via* p53, part of the transcriptome-wide consequences of their overexpression. In fact, the common dysregulated clusters we extracted as a result were far more informative as they were composed of meningioma-associated genes and pathways of regulation of apoptosis and of brain development. These processes all agree with the tumor suppressor potential of these miRs. Further in line with previous results, gene expression *via* the DREAM complex was again pointed out as a culprit in meningioma progression (12), which reinforces the usefulness of our miR-mimics signature as it was able to segregate samples according partly to this feature in external human datasets. Of note, in the landmark cohort from Clark et al. (14), POLR2A-mutated samples clustered outside the main subgroups and were mostly unresponsive to the signature, which delineates its direct implication in cell cycle progression and gene expression. In the larger 5-clusters signature, target genes of miR-16 and miR-519 were expectedly enriched, but were also targets of HuR, which our group reported as upregulated and as a poor prognosis factor in meningioma progression and recurrence (43). Here, HuR was strongly under-expressed following overexpression of both miRs, and part of the gene expression/cell cycle cluster. We propose thus that the interplay of the three markers, namely HuR, miR-16, and miR-519 is of importance in meningioma development and progression. In this regard, the restricted 208-gene signature deserves attention as many of these genes (**Table S8**) may emerge as precious additions to the meningioma biomarker repertoire. Moreover, many targets genes and signatures proposed by the transcriptomics (HuR, HIF1A and hypoxia, EP300) have already been functionally validated or meta-analytically cross-validated in previous works (11, 43, 61). In a previous study on HuR in meningioma patients and following knockdown in the same meningioma cell lines, we already correlated mRNA and protein levels, as well as HuR cellular localization and post-translational modifications. We also extensively studied HuR mRNA targets *via* transcriptomics (43). In the present work, we overlap the signatures obtained previously and the miR-mimic signatures. By doing so we recover the hypoxia signature that was functionally validated in HuR work, including its action on cell growth. Hypoxia being under the tight control of HIF1A, we demonstrated it to be a hallmark of meningioma progression. Concerning the transcription factor EP300, we previously correlated a methylation signature with grade, progression, and proliferation markers such as Ki-67 and MCM6 and showed that the regulatory regions associated with meningioma growth are highly enriched in CpG islands located in enhancers in distal regions (61). This methylation signature is known to be a mark of tissue-specific EP300 activity, and involved in cell growth and division in cancers (62, 63). In our final integrative experiment on human tissues, we provide the hint of a mechanistic link between the two miR expressions and that of hallmark target genes we report from our own transcriptomic findings: markers from each of the five clusters, miR-16 and/or miR-519 targets, and progression markers associated with meningioma aggressiveness. These results translate directly *in vivo*, onto meningioma samples of various histological subtypes and grades, therefore we believe our proposed biomarkers and signatures could have wide biological and clinical meaning.

In healthy tissues, little is known on the molecular differences between the arachnoid and the dura mater. Contrary to miR-16, here we observed similarly diminished miR-519 levels in the dura mater and in tumoral tissues. Further investigating the transcriptomic profiles available for healthy controls with and without the 208-gene signature uncovered a dura mater pattern overlapping with other embryologic tissues and an enrichment in miR-519 mRNA targets, and no overlap with the adult arachnoids, confirming our first observation on human meningiomas.

Decreased miR-16 levels have been observed in colorectal cancer (64), non-small cell lung carcinoma (24, 59, 65), chronic lymphocytic leukemia (66), pituitary adenomas (67), and gliomas (30). Our findings are consistent with these previous observations and suggest the involvement of miR-16 in tumor suppression. The molecular basis for the suppressive action in meningioma growth, however, is not clear. Yang et al. reported that miR-16 inhibits cell growth and reduces invasive properties in a glioma cell line through the suppression of BCL2 and NF-kappaB1/MMP-9 signaling pathway (30). Alternatively, miR-16 may mediate its action through the inhibition of FGF receptors or SMAD3 (68–70). MiR-16 may interact directly with HuR mRNA at its 3’UTR or with HuR protein itself. Indeed, Xu et al. showed that miR-16 decreases the expression of the pro-oncogenic HuR protein in breast cancer by inhibiting the translatability of its mRNA *via* direct interaction within the 3’UTR of HuR transcript (25). It is also compatible with an evidence in colon cancer cells indicating disrupted miR-16 binding to its cytoplasmic targets due to miR-16/HuR interaction. Incidentally, these competitive interactions are thought to occur in the cytoplasm as weaker association between HuR and miR-16 was noted when nucleocytoplasmic trafficking of HuR was inhibited (64).

Like miR-16, miR-519 has been linked to tumor suppression. Its downregulation has been reported in laryngeal squamous cell carcinoma (23), as well as ovary (45), lung (24), and kidney cancers (44). In several cancer cell lines (ovarian, colon, and laryngeal), miR-519 was shown to inhibit cell growth and proliferation, and, in animal model, the anti-tumorigenic properties of miR-519 were demonstrated in cultured HeLa cells xenografted in athymic mice (23, 44–46). Its mechanism of action may also be mediated through HuR as two miR-519 interaction sites have been evidenced within HuR mRNA: one within the coding region, and the other in the 3’UTR (45). Possibly, much like miR-16, miR-519 may alter HuR expression by inhibiting the translation of HuR mRNA (23, 45). MiR-519 may also exert its action *via* other signaling molecules independent of HuR. Abdelmohsen et al. identified numerous miR-519 targets in addition to HuR, *via* a combination of proteome, microarray, and miR-519-mRNA interaction analyses (71). They found that miR-519 inhibits the growth and survival of tumor cells *via* repressing the expression of proteins involved in DNA maintenance (including DUT1, EXO1, RPA2, and POLE4) and intracellular calcium homeostasis (ATP2C1 and ORAI1). In this work we report that miR-519 effects on cell growth are linked with transcriptomic programs related to cancer hallmarks such as the regulation of apoptosis and hypoxia pathways, in the fashion of what we observed with HuR activity (43).

In meningioma, one additional question concerns the upstream mechanisms leading to the downregulation of both miR-16 and miR-519. Chromosomal deletions at 13q14 have been linked to miR-16 downregulation in several hematological malignancies (66, 72). DNA methylation may also participate in the inhibition of certain miR-related gene transcription (73). For example, our group recently described methylation of miR-16-linked regulatory regions as being strongly correlated with proliferation markers and indices (61). It remains unclear, however, what causes the downregulation of miR-519 in meningiomas. Despite lacking a complete understanding of the transcriptional regulation of miR-16 and miR-519 and of their downstream effects, we investigated possible prognostic values of these miRs in meningiomas by searching for correlations between miR expression, WHO histological grade, and progression-free survival. We found no such correlation. Possible explanations include that these miRs participate in tumor formation during early stages, and that their expression levels, albeit high in normal tissue, decrease in tumors to levels close to the detection limit of the chosen assay method and are therefore difficult to quantify reliably. In other types of cancers, evidence suggests that miR-16 may be of prognostic value. For example, in colorectal cancer, the 5-year overall survival rate was significantly reduced for patients with lower miR-16 expression (67, 74). Also, in investigated T lymphoblastic lymphoma/acute lymphoblastic leukemia (T-LBL/ALL) lymph node samples, authors found evidence of improved overall 1-year survival rate for patients with higher miR-16 expression levels (75).

The use of chemotherapy as an additional treatment for patients with recurrent meningioma was considered by Balik et al. The authors showed that *in vitro* chemosensitivity was most effectively obtained with cisplatin (76), which was shown to inhibit cell proliferation *via* upregulation of miR-16 in neuroblastoma both *in vivo* and *in vitro* (77). Additionally, therapeutic applications of miRs represent a novel strategy to influence clinical outcomes in cancer patients. Fujita et al. reviewed the recent trials on small RNAs, focusing on the modulation of miR levels (18). Reid et al. demonstrated that the restoration of miR-16 levels results in inhibition of growth in malignant pleural mesothelioma *in vitro* and improves antimetabolite drug sensitivity, justifying the onset of phase I clinical trials (“MesomiR-1”, ClinicalTrials.gov identifier: NCT02369198) (60, 78). Here, we showed that miR-16 is an interesting candidate for miR replacement therapy in meningioma. Indeed, miR-16 experimental overexpression resulted in a significant decrease of cell growth, both in the anaplastic IOMM-Lee cell line and in the benign meningioma Ben-Men-1 cell line, showing significant effects on the cell cycle. We found that the inhibition of cell growth by miR-16 mimic is dose-dependent, this important pharmacologic property reinforcing its attractivity for therapeutic purpose. These first pre-clinical results need now to be validated with *in vivo* experimental studies. Similar to “TargomiRs”, which showed interesting preliminary results in mesothelioma (78), this miR could be specifically addressed to meningioma tumor cells through vectors loaded with miR-16 mimics and targeted to receptors specifically expressed by meningioma cells (e.g., SSTR2).

Some authors have argued about the meningothelial origin of IOMM-Lee cells and whether it can be considered a realistic model of meningioma (49, 79). While these cells demonstrate specific features of malignancy, here we make extensive use of this cell line and by doing so reviewed its molecular relevance as a high-grade anaplastic cell line. Apart for the fact that we needed a fully compatible model to link and overlap our results with what we previously validated on HuR and hypoxia, we persistently uncovered dysregulated meningioma signature genes, such as *CDKN1A*, *HIF1A*, *EGFR*, *MUC1 (*EMA*)*, *NRAS*, *MMP2*, *STAT3*, *ETV6*, *MN1*, *ERCC2*, *MDM2*, *NF2* and *TP53*, some of them well known to be often associated together in meningioma. In our opinion, the upregulation of many of these genes with the miR-mimics or HuR knockdown demonstrate a profile correlating with that of well-differentiated meningiomas. Furthermore, expression of proteins like SSTR2A (somatostatin receptor 2A), EMA (epithelial membrane antigen, *MUC1* gene) and PR (progesterone receptor) is known to fit with meningioma diagnosis. Indeed, the transcriptomes of IOMM-Lee cell line, whether in control, miR-mimic or siHuR, showed expression of respective corresponding genes *SSTR2*, *MUC1* and *PGR* above the median level, with levels more than 2-fold higher basal expression in every samples.

In conclusion, the present study provides the first evidence for the downregulation of both miR-16 and miR-519 in human meningioma. We show that the overexpression of these two miRs can independently inhibit meningioma cell growth. The data from the dose-response experiments reported here indicate that miR-16 exerts strong inhibitory effects against cell growth. We also uncover a highly specific transcriptomic signature of miR-16/miR-519-dysregulated genes, enriched in cell cycle genes and HuR targets, and confirmed on external datasets of human meningiomas, suggesting that the putative tumor suppressor effect of these miRs is mediated, at least in part, *via* HuR direct or indirect inhibition.

## Data availability statement

The datasets presented in this study can be found in online repositories. The names of the repository/repositories and accession number(s) can be found below: https://www.ncbi.nlm.nih.gov/geo/, GSE98848 (produced in this work), GSE95212, GSE85135, GSE126563, GSE50641, GSE74385, GSE88721.

## Ethics statement

The studies involving human participants were reviewed and approved by Institutional Review Board DC2008-459. The patients/participants provided their written informed consent to participate in this study.

## Author contributions

Conceptualization, SH, J-LG, J-MV, S-FB-H and GG; Data curation, J-MC; Formal analysis, SH, J-MC and GG; Funding acquisition, J-LG and GG; Investigation, DH, FR, LV, SH and GG; Methodology, SH, AO, DH, FR, LV, S-FB-H and GG; Project administration, J-LG and GG; Software, SH, AO and RH; Supervision, S-FB-H and GG; Writing – original draft, SH, J-MC, AO and GG; Writing – review & editing, SH, RH, J-MV, S-FB-H and GG. All authors contributed to the article and approved the submitted version.
