# Supplementary material for: MicroRNAs miR-16 and miR-519 control meningioma cell proliferation via overlapping transcriptomic programs shared with the RNA-binding protein HuR
Source: Front Oncol. 2023 Aug 2;13:1158773. doi: 10.3389/fonc.2023.1158773 (PMC10433742; doi:10.3389/fonc.2023.1158773)
Supplement: Supplementary file 1 [file DataSheet_1.pdf]

*Supplementary Material*

# MiR-16 and MiR-519 Suppress Tumor Cell Proliferation in Meningiomas via HuR Inhibition

**Sébastien Hergalant*, Jean-Matthieu Casse, Abderrahim Oussalah, Rémi Houlgatte, Déborah Helle, Fabien Rech, Laurent Vallar, Jean-Louis Guéant, Jean-Michel Vignaud, Shyue-Fang Battaglia-Hsu and Guillaume Gauchotte***

*** Correspondence:**
Corresponding Authors: sebastien.hergalant@univ-lorraine.fr g.gauchotte@chru-nancy.fr

## Supplementary Figures

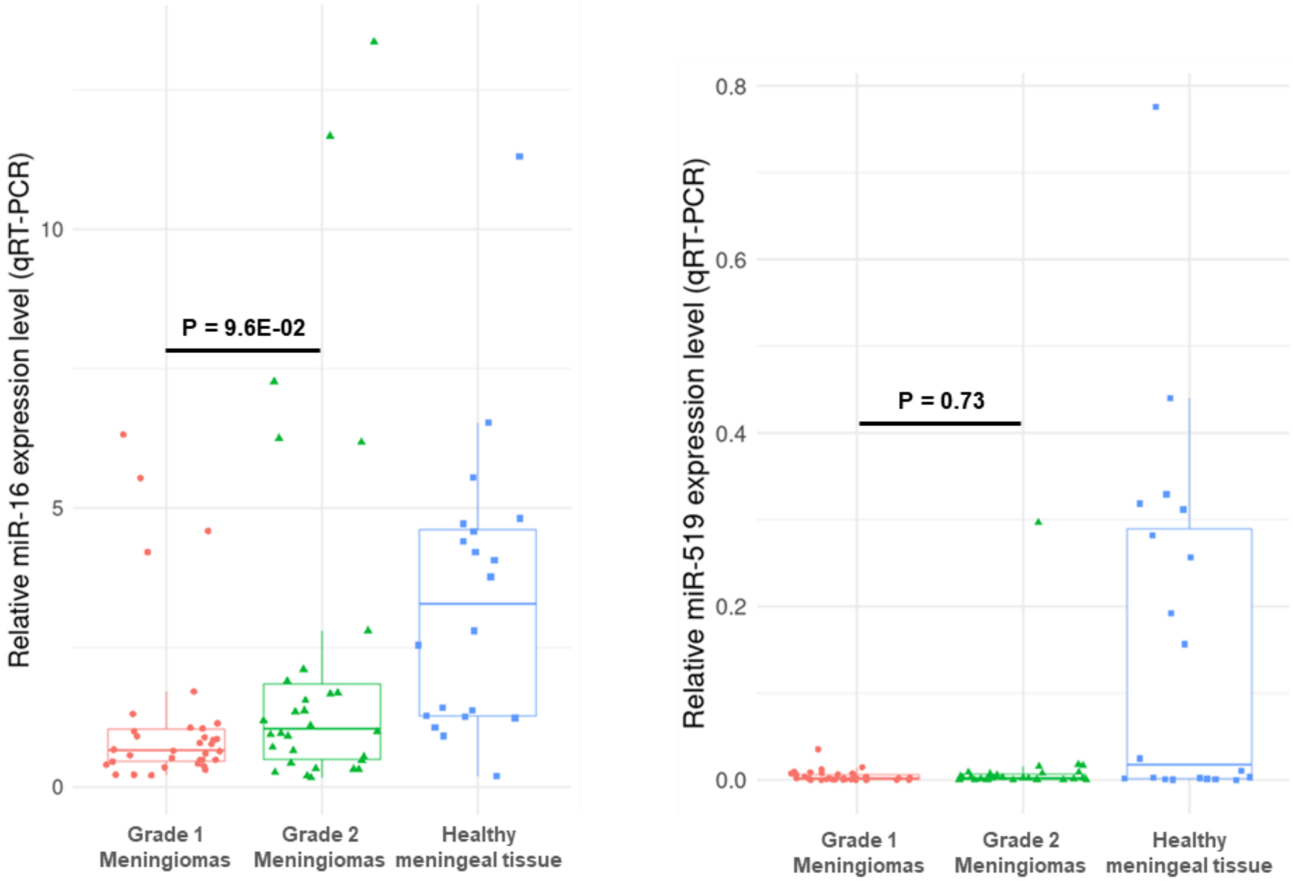


**Supplementary Figure S1.** MiR-16 and miR-519 expression levels in human meningioma and healthy meningeal tissues. Quantitative reverse transcriptase polymerase chain reaction (qRT-PCR) analysis of miR-16 (A) and miR-519 (B) expression in human meningiomas (grade 1: n = 30; grade 2: n = 34) and non-tumoral tissue (n = 20). Boxplot (minimum, first quartile, median, third quartile, and maximum) with individual scatterplots. Wilcoxon Mann-Whitney U-tests.

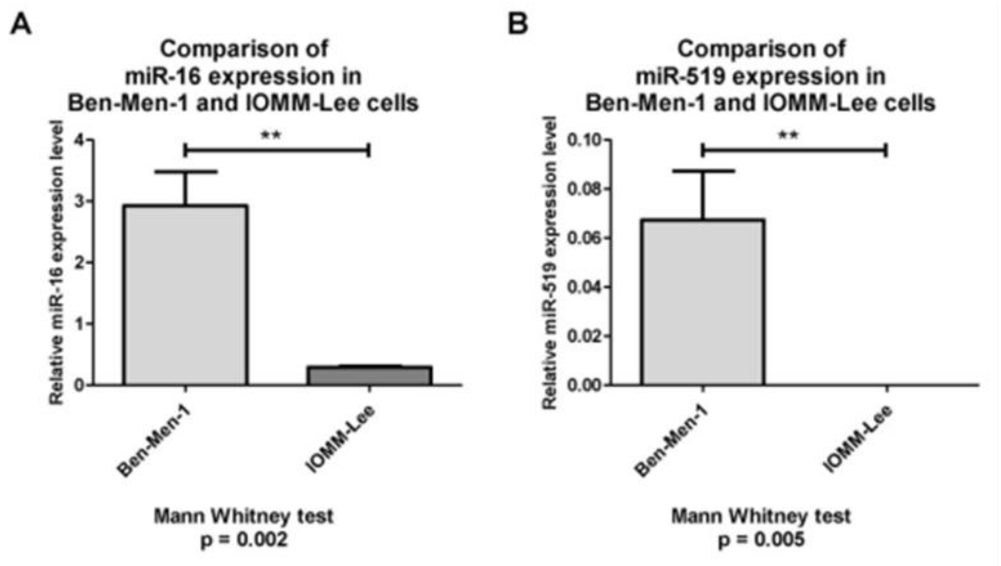


**Supplementary Figure S2.** Comparison of basal expression level of miR-16 and miR-519 between the human benign meningioma immortalized Ben-Men-1 cell line and the human anaplastic meningioma IOMM-Lee cell line, showing a significantly lower expression of miR-16 (A) and miR-519 (B) in the IOMM-Lee cells (qRT-PCR; n=6).

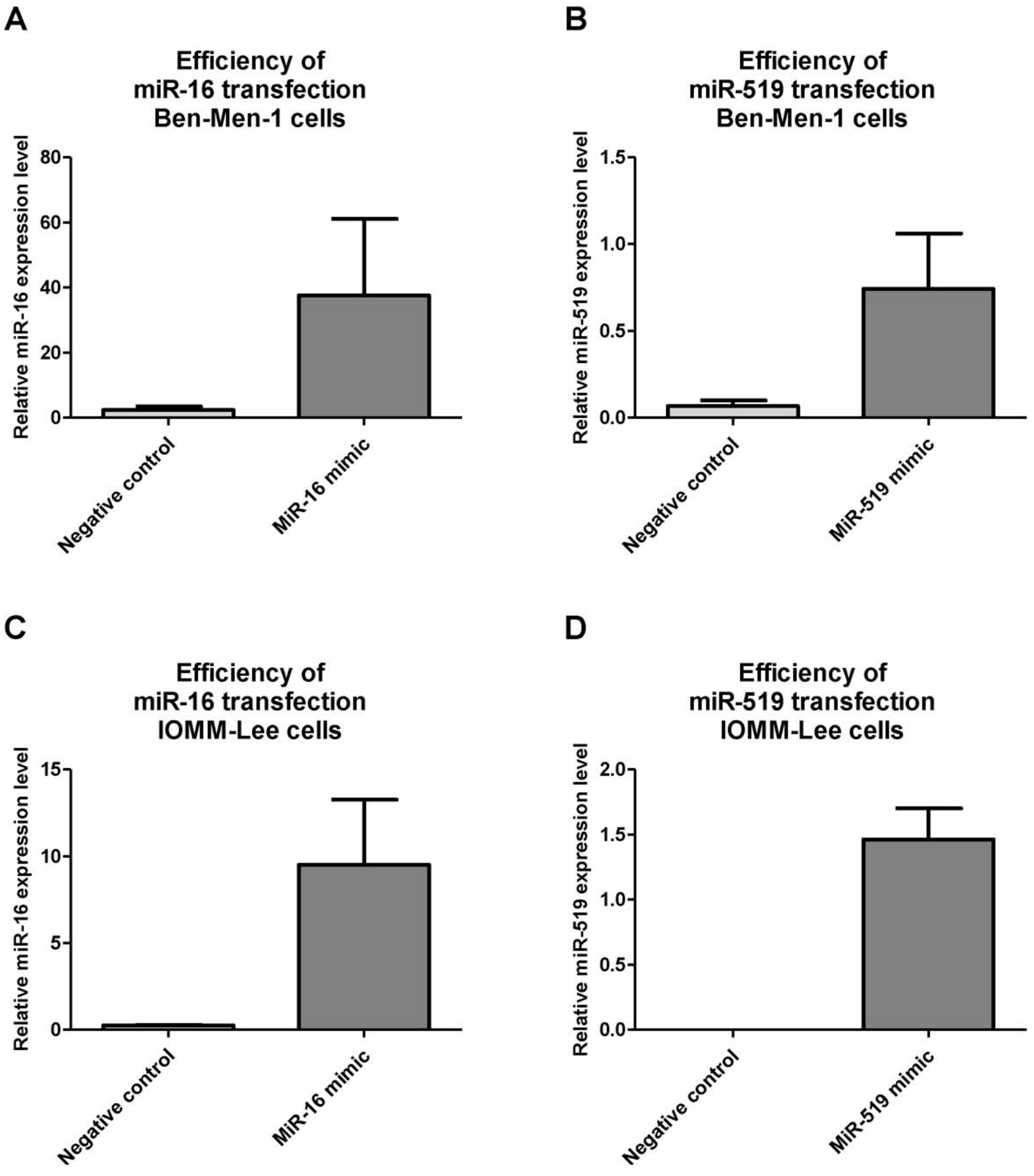


**Supplementary Figure S3.** Efficiency of miR mimics transfection evaluated by qRT-PCR against miR-mimic negative controls. (A) Transfection of hsa-miR16 mimic in Ben-Men-1 cell line. (B) Transfection of hsa-miR519 mimic in Ben-Men-1 cell line. (C) Transfection of hsa-miR16 mimic, IOMM-Lee cell line. (D) Transfection of hsa-miR519 mimic in IOMM-Lee cell line (n=3).

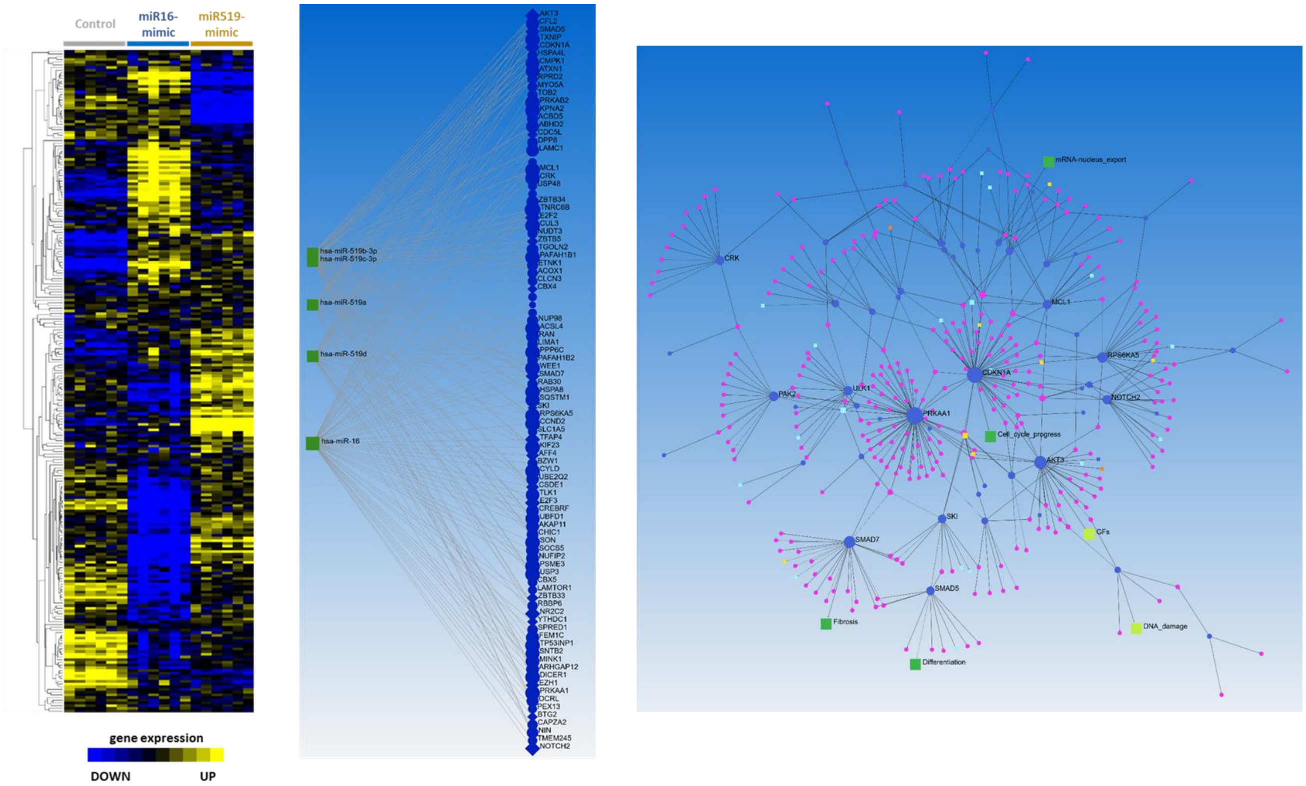


**Supplementary Figure S4.** Gene signature identified with miR-16 and miR-519 transcriptomics. Left panel: Hierarchical clustering heat map of the 208 genes from the five differential clusters (c1 to c5) further reduced to mir-16, miR-519 overlapping targets. The highly differential and correlative structure is representative of the five clusters. Middle panel: miR network of shared targets of miR-16 and miR-519. Squares: miRs, losanges: transcription factors / transcriptional regulators; circle: other targets). Shape size is proportionally linked with the number of shared miRs. Right panel: Signaling network reconstructed with the 208-gene signature identified with miR-16 and miR-519 transcriptomics. Circles (pink and blue): gene targets, blue squares: chemicals, green squares (light and dark): stimuli and phenotypes (ontologies), turquoise and yellow squares: protein complexes and families, orange squares: other small molecules. Networks were constructed with NetworkAnalyst (https://www.networkanalyst.ca/).

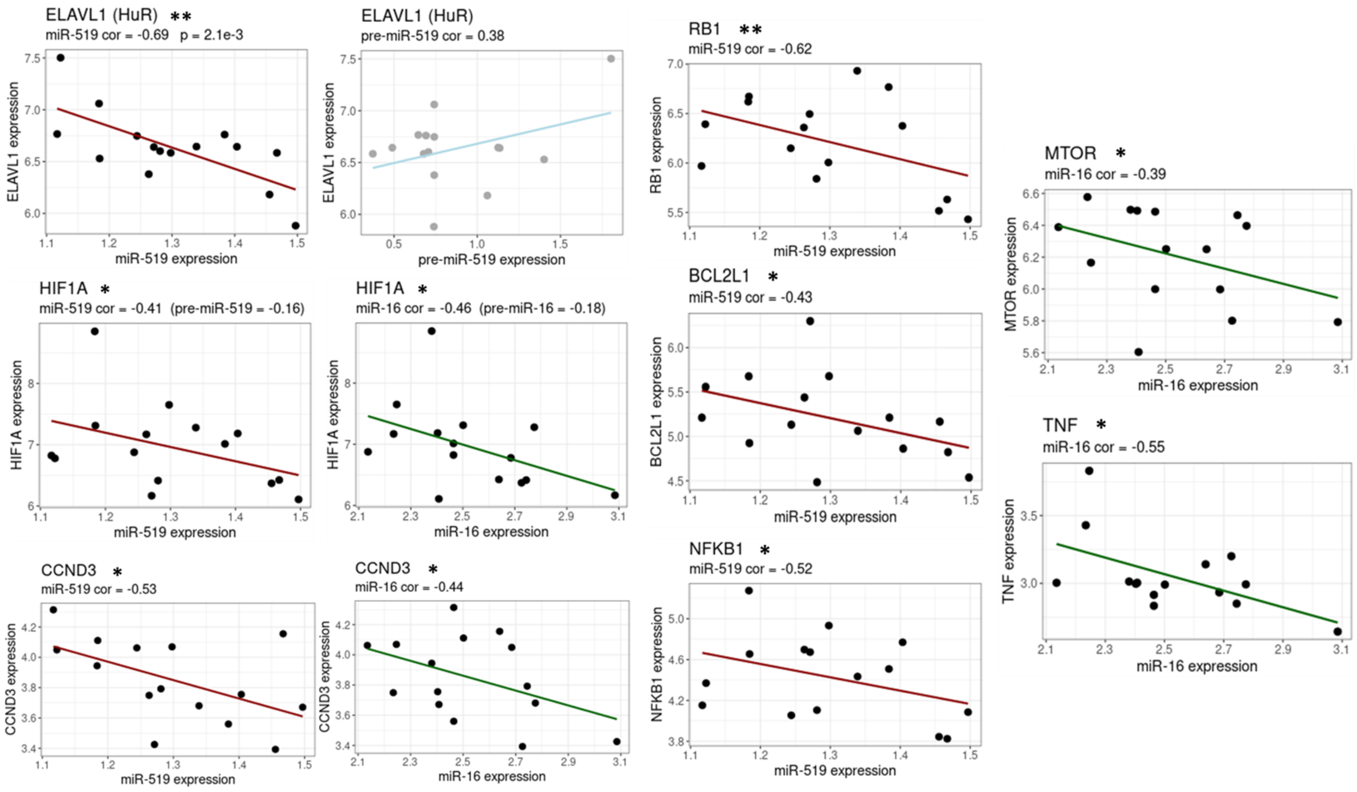


**Supplementary Figure S5.** Validation of main transcriptomic results through the integration of meningioma patient transcriptomes and corresponding microRNA microarrays (public dataset GSE88721 from Dalan et al, 2017; PMID: 28327132; https://www.ncbi.nlm.nih.gov/geo/query/acc.cgi?acc=GSE88721). Hallmark genes from the original 5-cluster transcriptomic signature, the meningioma signature and proposed progression markers are tested against candidate miR-16 and miR-519 and preprocessed pre-miR-16 and pre-miR519 (Pearson's correlations; * $p < 0.05$; ** $p < 0.01$), including direct miR-16 and/or miR-519 target RNAs. Patient samples include diverse histological subtypes (including one healthy meninges) and meningioma grades 1 and 2. Expression levels are normalized against background and log2-transformed.
